# Sexual quality of life of adolescents and young adult breast cancer survivors

**DOI:** 10.1016/j.esmoop.2024.102234

**Published:** 2024-01-27

**Authors:** N.J.M.C. Vrancken Peeters, C. Vlooswijk, R.M. Bijlsma, S.E.J. Kaal, J.M. Kerst, J.M. Tromp, M.E.M.M. Bos, T. van der Hulle, R.I. Lalisang, J. Nuver, M.C.M. Kouwenhoven, I.M.C. van der Ploeg, W.T.A. van der Graaf, O. Husson

**Affiliations:** 1 Department of Medical Oncology, Netherlands Cancer Institute-Antoni van Leeuwenhoek, Amsterdam; 2 Department of Plastic and Reconstructive Surgery, Erasmus MC Cancer Institute, Erasmus University Medical Centre, Rotterdam; 3 Research and Development, Netherlands Comprehensive Cancer Organisation, Utrecht; 4 Department of Medical Oncology, University Medical Centre, Utrecht; 5 Department of Medical Oncology, Radboud University Medical Centre, Nijmegen; 6 Department of Medical Oncology, Amsterdam University Medical Centres, Amsterdam; 7 Department of Medical Oncology, Erasmus MC Cancer Institute, Erasmus University Medical Centre, Rotterdam; 8 Department of Medical Oncology, Leiden University Medical Centre, Leiden; 9 Department of Internal Medicine, Division of Medical Oncology, GROW-School of Oncology and Reproduction, Maastricht UMC+ Comprehensive Cancer Centre, Maastricht; 10 Department of Medical Oncology, University Medical Centre Groningen, Groningen; 11 Department of Neurology, Amsterdam UMC, Amsterdam University Medical Centres, Amsterdam; 12 Department of Surgical Oncology, Netherlands Cancer Institute-Antoni van Leeuwenhoek, Amsterdam; 13 Department of Psychosocial Research and Epidemiology, Netherlands Cancer Institute, Amsterdam; 14 Department of Surgical Oncology, Erasmus MC Cancer Institute, Erasmus University Medical Centre, Rotterdam, The Netherlands

**Keywords:** adolescents and young adults (AYAs), breast cancer, health-related quality of life (HRQoL), sexual quality of life (QoL), survivorship

## Abstract

**Background:**

With increasing survival rates of adolescents and young adults (AYAs) with breast cancer, health-related quality of life (HRQoL) becomes more important. An important aspect of HRQoL is sexual QoL. This study examined long-term sexual QoL of AYA breast cancer survivors, compared sexual QoL scores with that of other AYA cancer survivors, and identified factors associated with long-term sexual QoL of AYA breast cancer survivors.

**Materials and methods:**

Data of the SURVAYA study were utilized for secondary analyses. Sexual QoL was assessed using the European Organization for Research and Treatment of Cancer Quality of Life cancer survivorship core questionnaire (EORTC QLQ-SURV100). Descriptive statistics were used to describe sexual QoL of AYA cancer survivors. Linear regression models were constructed to examine the effect of cancer type on sexual QoL and to identify factors associated with sexual QoL.

**Results:**

Of the 4010 AYA cancer survivors, 944 had breast cancer. Mean sexual QoL scores of AYA breast cancer survivors ranged from 34.5 to 60.0 for functional domains and from 25.2 to 41.5 for symptom-orientated domains. AYA breast cancer survivors reported significantly lower sexual QoL compared to AYA survivors of other cancer types on all domains. Age, time since diagnosis, relationship status, educational level, chemotherapy, hormonal therapy, breast surgery, body image, and coping were associated with sexual QoL of AYA breast cancer survivors.

**Conclusions:**

AYA breast cancer survivors experience decreased sexual QoL in the long term (5-20 years) after diagnosis and worse score compared to AYA survivors of other cancer types, indicating a clear need to invest in supportive care interventions for those at risk, to enhance sexual well-being.

## Highlights

• AYA breast cancer survivors experience significantly lower long-term sexual QoL compared to survivors of other cancer types.
• Age, time, relationship status, education, treatment and psychosocial factors are associated with sexual QoL.
• Investment in supportive care interventions can enhance sexual QoL of AYA breast cancer survivors in the future.

## Introduction

Adolescents and young adults (AYAs) are defined by the US National Cancer Institute as patients aged between 15 and 39 years at the time of their initial cancer diagnosis; however, this definition can be flexibly applied depending on the health care system.^1^^,^^2^ In the Netherlands, AYAs are categorized as patients aged between 18 and 39 years at the time of their diagnosis.^2^ The incidence of cancer has increased within this population in the last decades.^3^^,^^4^ Among female AYA cancer patients, breast cancer is one of the most common cancer types.^4^, ^5^, ^6^ Advancements in early detection and cancer treatment have contributed to the increased survival for (AYA) cancer patients, making long-term health-related quality of life (HRQoL) after diagnosis and treatment more important.^4^^,^^7^, ^8^, ^9^, ^10^, ^11^, ^12^

A highly important aspect of overall HRQoL is sexual health.^13^^,^^14^ Sexual health is defined as a state of physical, emotional, mental, and social well-being in relation to sexuality and not merely the absence of disease, dysfunction, or infirmity.^15^ Both the cancer diagnosis and/or the corresponding treatment can have significant impacts on sexual health, leading to negative effects on overall HRQoL.^16^, ^17^, ^18^, ^19^, ^20^, ^21^ The type of cancer plays an important role in sexual health, as especially, women with gynaecological or breast cancer are at high risk for sexual dysfunction, including, for example, problems with sexual arousal and desire or pain.^17^^,^^22^, ^23^, ^24^ Previous research demonstrated that sexual health of women with breast cancer is significantly worse compared to that of women from the general population.^25^ Especially young breast cancer survivors suffer from negative effects on sexual health, with sexual dysfunction being highly prevalent in this population.^26^, ^27^, ^28^, ^29^, ^30^, ^31^, ^32^, ^33^

Although sexual health plays an important role in HRQoL of AYA cancer survivors, it is often inadequately addressed by health care professionals.^16^^,^^27^^,^^34^, ^35^, ^36^ Furthermore, it is known from previously published literature that AYA cancer survivors experience ongoing problems with sexual functioning up to 2 years after their initial diagnosis.^37^ Unfortunately, there is limited knowledge regarding long-term (>5 years after diagnosis) sexual health of AYA cancer survivors. Moreover, most current studies have primarily focused on sexual dysfunction rather than also focusing on sexual QoL and well-being.^29^^,^^33^^,^^37^, ^38^, ^39^ Therefore, the primary aim of this study is to examine long-term sexual QoL (as the mean denominator of sexual health) of AYA breast cancer survivors and compare it with that of (female) AYA survivors of other cancer types. Additionally, this study aims to identify factors associated with long-term sexual QoL of AYA breast cancer survivors.

## Materials and methods

### Data

Data of the (HRQoL and late effects among SURVivors of cancer in AYA) SURVAYA study was utilized for secondary analyses.^2^ The SURVAYA study is an observational population-based, cross-sectional cohort study among 5- to 20-year survivors of AYA cancer (18-39 years at time of diagnosis). The aim of this study was to gain more insight into subgroups of AYA cancer survivors at risk for long-term health problems. The study was conducted in eight university medical centres and one cancer-specific hospital in the Netherlands. The Netherlands Cancer Registry (NCR) was used to select the AYA cancer survivors from the participating cancer centres. The NCR collects detailed data (disease and treatment characteristics) on cancer patients in the Netherlands.^40^ The PROFILES (Patient Reported Outcomes Following Initial treatment and Long-term Evaluation of Survivorship) registry was used for the collection of patient-reported outcomes.^41^ The questionnaire data of the SURVAYA study collected via PROFILES were merged with the clinical cancer data (such as tumour and treatment characteristics) of the NCR at the end of the study. More detailed information about the data collection of the SURVAYA study has previously been published elsewhere.^2^

Informed consent was obtained from all subjects (responders) involved in the study. The SURVAYA study was conducted in accordance with the Declaration of Helsinki and approved by the Netherlands Cancer Institute Institutional Review Board (IRB-IRBd18122) on 6 February 2019.

### Outcome measure

Sexual QoL was assessed using the European Organization for Research and Treatment of Cancer Quality of Life cancer survivorship core questionnaire (EORTC QLQ-SURV100).^2^^,^^42^^,^^43^ The EORTC QLQ-SUV100 is developed to evaluate HRQoL of disease-free cancer survivors. The EORTC QLQ-SURV100 includes 100 items and different scales regarding symptoms, functioning, financial difficulties, and global health. Each item is scored on a four-point Likert scale ranging from 1 (not at all) to 4 (very much), with additional ‘not applicable’ and ‘I don’t want to say’ options. After conducting linear transformation, all scales and single items range from 0 to 100. For functional scales, a higher score indicates a higher QoL, while for the symptom-orientated scales a higher score indicates more severe symptoms. Eight items (S101-S105 and S107-S109) are related to (female) sexual QoL (sexual problems and functioning) and were analysed in this study. Of the eight items, six can be combined into three overarching scales, namely sexual problems (S104 and S107), sexual functioning (S108 and S109), and sexual problems when sexually active (S101 and S103). The remaining two items are covered by the questions: ‘has sexual activity been enjoyable for you (S102)’ and, for women only, ‘have you experienced a dry vagina during sexual activity (S105)’ (Supplementary Appendix A, Table S1, available at https://doi.org/10.1016/j.esmoop.2024.102234).

All items could be answered independently of each other, which may lead to variations in response percentages per item.

### Covariates

Patient demographics, primary treatment details, and tumour characteristics were obtained from the SURVAYA study and NCR. The included variables of interest were age at diagnosis, time since diagnosis, body mass index, educational level, relationship status, chemotherapy, hormonal therapy, radiotherapy, type of breast surgery consisting of breast-conserving surgery (BCS) with or without an axillary lymph node dissection (ALND) and mastectomy with or without an ALND, body image, and coping mechanism (maladaptive and adaptive). Variables were chosen based on previously published literature.^24^^,^^33^^,^^38^^,^^39^^,^^44^^,^^45^

Body image is a functional scale of the EORTC QLQ-SURV100 and consists of two single items (S39 and S40).

To measure the cognitive coping mechanism, the Cognitive Emotion Regulation Questionnaire (CERQ) was used.^46^ The CERQ consists of nine scales (self-blame, other-blame, rumination, catastrophizing, positive refocusing, planning, positive reappraisal, putting into perspective, and acceptance). The full CERQ comprises four items for each scale; however, in the current study the shortened version of two items was used. The answer options for each item can range from 1 [(almost) never] to 5 [(almost) always]. A total score for each scale can be calculated by summing the scores of the two corresponding items (range: 2-10). A higher scale score indicates a greater level of that specific cognitive emotion regulation strategy.^46^^,^^47^ Based on these scales a maladaptive scale consisting of self-blame, other-blame, rumination, and catastrophizing, and an adaptive scale consisting of positive refocusing, planning, positive reappraisal, putting into perspective, and acceptance were constructed. Consequently, the maladaptive scale ranges from 8 to 40 and the adaptive scale ranges from 10 to 50.

### Data analysis

Descriptive statistics were used to describe patient tumour and treatment characteristics. Depending on the nature of the variable, *t*-tests (continuous variables) or chi-square tests (categorical variables) were used to compare the baseline characteristics between AYA breast cancer survivors with that of AYA survivors of other cancer types.

Bar charts were constructed for each of the eight sexual QoL questions of the EORTC QLQ-SURV100 to examine and visualize long-term sexual QoL of AYA breast cancer survivors and AYA survivors of other cancer types.

Linear regression models of the three scales and two single items of sexual QoL were constructed to examine the effect of cancer type on sexual QoL while correcting for time since diagnosis. To avoid multicollinearity, demographic, treatment, and tumour characteristics were not included in the models. AYA breast cancer survivors were chosen as the reference category. Additionally, a sensitivity analysis including only female AYA cancer survivors was conducted.

To identify potential factors associated with long-term sexual QoL of AYA breast cancer, five multiple linear regression models were constructed for each of the sexual QoL outcomes (ranging from 0 to 100).

Normality and homoscedasticity of the residuals were tested with residual plots. A two-sided *P* value of 5% was considered statistically significant and all statistical analyses were carried out using R statistical software (version 4.4.2).^48^

### Sample size calculation

As this study is a secondary analysis of data from the SURVAYA study, a sample size calculation is not applicable in this context. Details on the power analysis can be found in the SURVAYA study protocol.^2^ Given the limited number of missingness, a complete case analysis was carried out.

## Results

### Patient characteristics

In total, 4010 AYA cancer survivors (18-39 years at the time of diagnosis) were available from the SURVAYA database of which 944 (23.5%) were breast cancer survivors and 3066 (76.5%) were survivors of other cancer types (Table 1).

**Table 1:** Baseline characteristics of study population

	Breast cancer (*N* = 944)	Other cancer (*N* = 3066)	*P* value
Sex, *n* (%)			
Male	0 (0%)	1549 (50.5%)	—
Female	944 (100%)	1517 (49.5%)	
Age at diagnosis in years			
Mean (SD)	34.7 (3.85)	30.6 (6.07)	**<0.001**
Median (min-max)	36.0 (18.0-39.0)	31.5 (18.0-39.0)	
Time since diagnosis in years			
Mean (SD)	12.2 (4.52)	12.5 (4.50)	0.115
Median (min-max)	12.2 (4.41-21.6)	12.4 (4.45-22.1)	
BMI, *n* (%)			
Normal weight	573 (60.7%)	1614 (52.6%)	**<0.001**
Overweight	354 (37.5%)	1415 (46.2%)	
Missing	17 (1.8%)	37 (1.2%)	
Education, *n* (%)			
Low, no primary school	5 (0.5%)	23 (0.8%)	0.061
Intermediate, secondary education	377 (39.9%)	1345 (43.9%)	
High, college university	562 (59.5%)	1690 (55.1%)	
Missing	0 (0%)	8 (0.3%)	
Relationship status, *n* (%)			
Married/registered partnership	545 (57.7%)	1670 (54.5%)	0.190
Relationship	247 (26.2%)	871 (28.4%)	
Single	147 (15.6%)	514 (16.8%)	
Missing	5 (0.5%)	11 (0.4%)	
Smoking status, *n* (%)			
Never	511 (54.1%)	1744 (56.9%)	**0.031**
Former	359 (38.0%)	1029 (33.6%)	
Current	71 (7.5%)	276 (9.0%)	
Missing	3 (0.3%)	17 (0.6%)	
Chemotherapy, *n* (%)			
No	137 (14.5%)	1630 (53.2%)	**<0.001**
Yes	807 (85.5%)	1432 (46.7%)	
Missing	0 (0%)	4 (0.1%)	
Hormonal therapy, *n* (%)			
No	465 (49.3%)	3057 (99.7%)	**<0.001**
Yes	479 (50.7%)	5 (0.2%)	
Missing	0 (0%)	4 (0.1%)	
Radiotherapy, *n* (%)			
No	214 (22.7%)	1890 (61.6%)	**<0.001**
Yes	730 (77.3%)	1172 (38.2%)	
Missing	0 (0%)	4 (0.1%)	
Targeted therapy, *n* (%)			
No	771 (81.7%)	2927 (95.5%)	**<0.001**
Yes	173 (18.3%)	135 (4.4%)	
Missing	0 (0%)	4 (0.1%)	
Type of breast surgery, *n* (%)			
BCS	369 (39.1%)	—	—
BCS + ALND	133 (14.1%)	—	
Mastectomy	216 (22.9%)	—	
Mastectomy + ALND	219 (23.2%)	—	
Missing	7 (0.7%)	—	
Cancer type, *n* (%)			
Breast	944 (100%)	0 (0%)	—
Head and neck	—	124 (4.0%)	
Digestive tract, other	—	31 (1.0%)	
Colon and rectal	—	82 (2.7%)	
Bone, articular cartilage, and soft-tissue sarcomas	—	172 (5.6%)	
Respiratory	—	30 (1.0%)	
Melanoma	—	290 (9.5%)	
Other	—	11 (0.4%)	
Germ cell tumours	—	692 (22. 6%)	
Female genitalia	—	445 (14.5%)	
Male genitalia	—	6 (0.2%)	
Urinary tract	—	46 (1.5%)	
Lymphoid haematological malignancies	—	591 (19.3%)	
Myeloid haematological malignancies	—	148 (4.8%)	
Thyroid gland	—	248 (8.1%)	
Central nervous system	—	150 (4.9%)	
Tumour stage, *n* (%)			
1	338 (35.8%)	1388 (45.3%)	**<0.001**
2	447 (47.4%)	616 (20.1%)	
3	153 (16.2%)	420 (13.7%)	
4	6 (0.6%)	173 (5.6%)	
Missing	0 (0%)	469 (15.3%)	
Body image			
Mean (SD)	72.0 (25.7)	79.4 (24.2)	**<0.001**
Median (min-max)	83.3 (0-100)	83.3 (0-100)	
Missing, *n* (%)	59 (6.3%)	216 (7.0%)	
Maladaptive scale			
Mean (SD)	13.1 (3.60)	13.2 (3.91)	0.854
Median (min-max)	13.0 (8.00-27.0)	12.0 (8.00-40.0)	
Missing, *n* (%)	72 (7.6%)	230 (7.5%)	
Adaptive scale			
Mean (SD)	29.9 (7.18)	29.1 (7.50)	**0.002**
Median (min-max)	30.0 (10.0-50.0)	29.0 (10.0-50.0)	
Missing, *n* (%)	75 (7.9%)	240 (7.8%)	

Bold indicates *P* < 0.05.

ALND, axillary lymph node dissection; BCS, breast-conserving surgery; BMI, body mass index; SD, standard deviation.

### Long-term sexual QoL among AYA cancer survivors—individual questions

**Figure 1:**
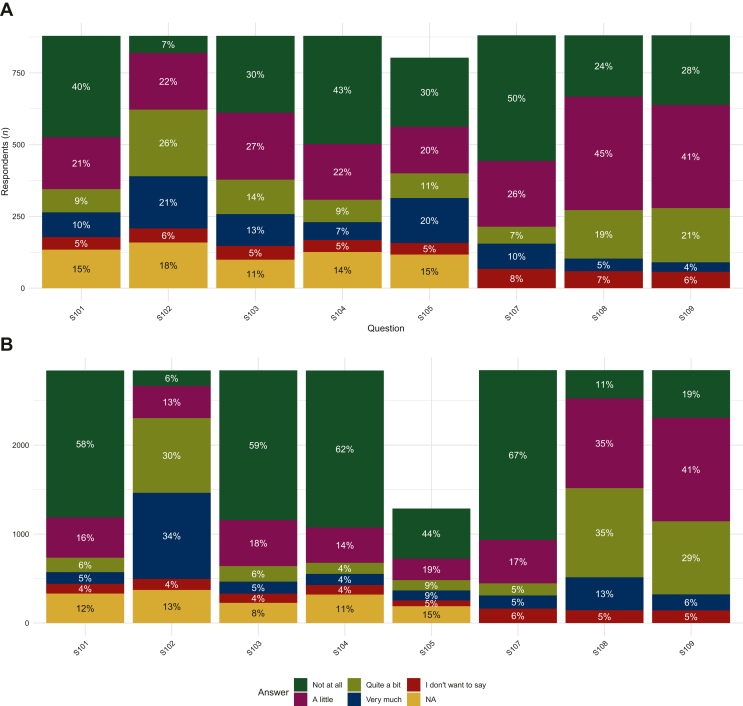
Long-term sexual QoL of AYA cancer survivors; individual questions. Bar charts of EORTC QLQ-SURV100 questions of *AYA breast cancer survivors (n)* (A) and *AYA survivors of other cancer types (n)* (B). S101: Have you had problems being sexually intimate? S102: Has sexual activity been enjoyable for you? S103: Have you had problems becoming sexually aroused? S104: Have you felt uncomfortable about the idea of being sexually intimate? S105 (women only): Have you experienced a dry vagina during sexual activity? S107: Have you avoided having sex? S108: Have you been interested in sex? S109: Have you been sexually active? AYA, adolescents and young adult; EORTC QLQ-SURV100, European Organization for Research and Treatment of Cancer Quality of Life cancer survivorship core questionnaire.

Figure 1 presents the responses to the individual long-term sexual QoL questions for AYA breast cancer survivors (Figure 1A) and for AYA survivors of other cancer types (Figure 1B). AYA breast cancer survivors tended to experience less sexual enjoyment and functioning compared to AYA survivors of other cancer types. In addition, AYA breast cancer survivors tended to experience more sexual symptoms and problems than AYA survivors of other cancer types.

### Long-term sexual QoL scores of AYA cancer survivors

Mean and median sexual QoL scores (0-100) of the three scales and two single items are presented in Table 2. AYA breast cancer survivors reported more sexual problems and symptoms and less sexual functioning compared to AYA survivors of other cancer types.

**Table 2:** Long-term sexual QoL scores

Sexual QoL	Breast cancer (*N* = 944)	Other cancer (*N* = 3066)
Has sexual activity been enjoyable for you? (0-100)		
Mean (SD)	60.0 (31.3)	70.3 (30.8)
Median (min-max)	66.7 (0-100)	66.7 (0-100)
Missing, *n* (%)	273 (28.9%)	722 (23.5%)
Have you experienced a dry vagina during sexual activity? (0-100, women only)		
Mean (SD)	41.5 (39.7)	26.1 (34.1)
Median (min-max)	33.3 (0-100)	0 (0-100)
Missing, *n* (%)	298 (31.6%)	2033 (66.3%)
Sexual problems (symptom scale) (0-100)		
Mean (SD)	25.2 (30.2)	15.1 (25.7)
Median (min-max)	16.7 (0-100)	0 (0-100)
Missing, *n* (%)	121 (12.8%)	356 (11.6%)
Sexual functioning (functional scale) (0-100)		
Mean (SD)	34.5 (25.4)	46.1 (25.8)
Median (min-max)	33.3 (0-100)	50.0 (0-100)
Missing, *n* (%)	118 (12.5%)	359 (11.7%)
Sexual problems when sexually active (symptom scale) (0-100)		
Mean (SD)	33.3 (32.5)	16.6 (25.4)
Median (min-max)	33.3 (0-100)	0 (0-100)
Missing, *n* (%)	202 (21.4%)	530 (17.3%)

For functional scales a higher score indicates greater QoL, and for symptom-orientated scales a higher score indicates more severe symptoms.

QoL, quality of life; SD, standard deviation.

### Effect of cancer type on long-term sexual QoL scores of AYA cancer survivors

**Figure 2:**
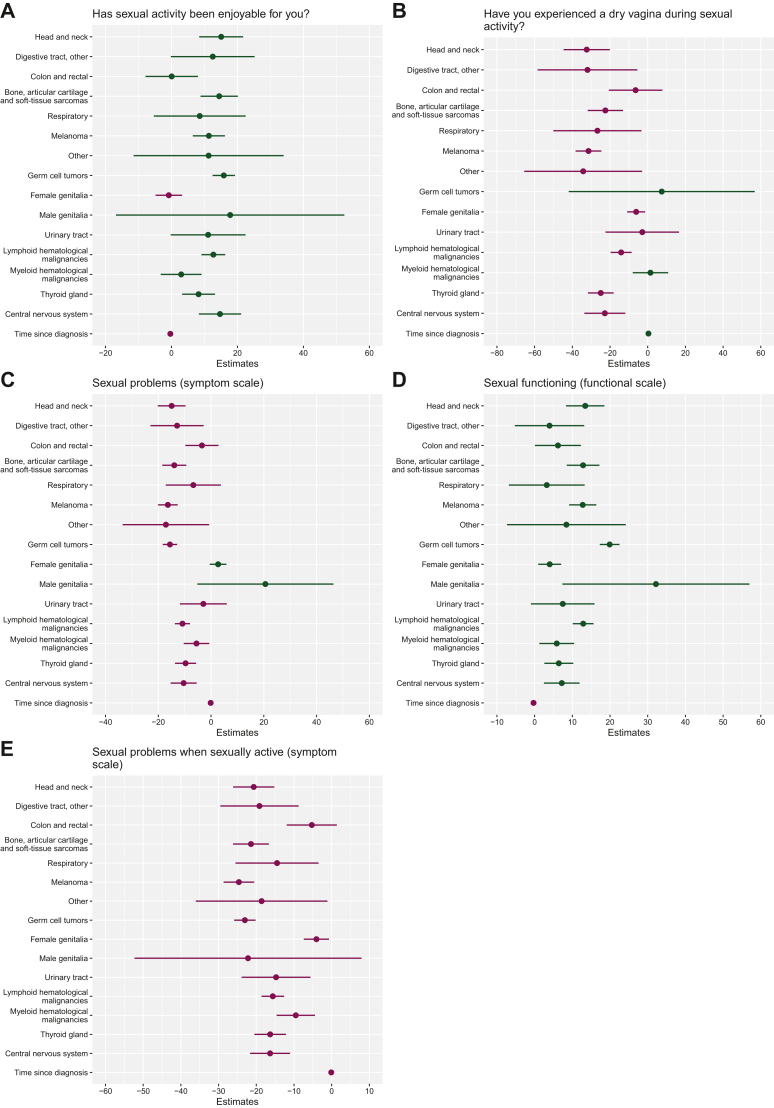
Regression models for the comparison of sexual QoL between AYA breast cancer survivors and AYA survivors of other cancer types. For functional scales, higher scores represent higher QoL; for symptom scales, higher scores indicate more severe symptoms. Green represents an increase in scores and purple, a decrease in scores. AYA breast cancer survivors were chosen as the reference category. AYA, adolescents and young adult; QoL, quality of life.

AYA breast cancer survivors scored significantly lower on sexual enjoyment and functioning compared to AYA survivors of various other cancer types (Figure 2A and D). In addition, AYA survivors of other cancer types experienced significantly less sexual symptoms compared to AYA breast cancer survivors (Figure 2B, C and E).

### Effect of cancer type on long-term sexual QoL scores of female AYA cancer survivors

**Figure 3:**
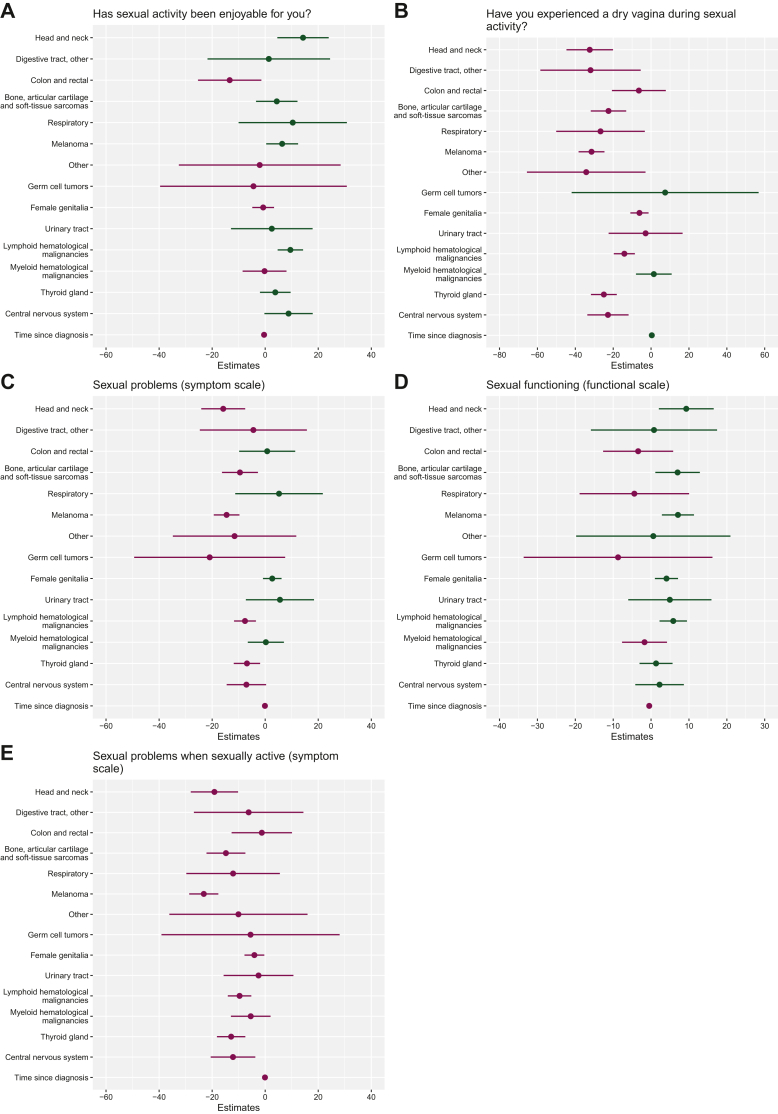
Regression models for the comparison of sexual QoL between AYA breast cancer survivors and female AYA survivors of other cancer types. For functional scales, higher scores represent higher QoL; for symptom scales, higher scores indicate more severe symptoms. Green represents an increase in scores and purple, a decrease in scores. AYA breast cancer survivors were chosen as the reference category. AYA, adolescents and young adult; QoL, quality of life.

For the sensitivity analysis including only female AYA cancer survivors, the results can be found in Figure 3. AYA breast cancer survivors scored significantly lower on sexual enjoyment and functioning (Figure 3A and D) and experienced significantly more sexual symptoms compared to female survivors of other cancer types (Figure 3B, C and E), except for female AYA survivors of colon and rectal cancer (Figure 3A).

### Factors associated with long-term sexual QoL of AYA breast cancer survivors

Older age at diagnosis, a longer time since diagnosis, a high or low education level compared to a medium educational level, receiving chemotherapy and/or hormonal therapy, and maladaptive coping were negatively associated with sexual QoL of AYA breast cancer survivors (Table 3).

**Table 3:** Multiple linear regression models of sexual QoL scores of AYA breast cancer survivors

Variables	Has sexual activity been enjoyable for you?		Have you experienced a dry vagina during sexual activity?		Sexual problems (symptom scale)		Sexual functioning (functional scale)		Sexual problems when sexually active (symptom scale)	
	Estimates	*P*	Estimates	*P*	Estimates	*P*	Estimates	*P*	Estimates	*P*
Intercept	92.16	**<0.001**	−0.22	0.991	7.42	0.556	44.84	**<0.001**	20.93	0.139
Age at diagnosis	−1.23	**<0.001**	1.49	**<0.001**	0.86	**0.001**	−0.82	**<0.001**	1.04	**<0.001**
Time since diagnosis	−0.20	0.494	0.59	0.105	0.02	0.942	−0.55	**0.008**	−0.03	0.896
BMI										
Normal weight	Reference		Reference		Reference		Reference		Reference	
Overweight	0.09	0.972	−2.93	0.370	1.39	0.512	−0.40	0.828	−0.87	0.715
*Education*										
Intermediate, secondary education	Reference		Reference		Reference		Reference		Reference	
High, college university	−2.21	0.374	2.24	0.478	4.51	**0.028**	−1.55	0.385	3.49	0.130
Low, no primary school	−32.65	0.127	77.62	**0.039**	41.41	**0.035**	−28.96	0.089	−10.01	0.633
Relationship status										
Single	Reference		Reference		Reference		Reference		Reference	
Married/registered partnership	−8.57	**0.042**	3.37	0.514	−1.58	0.587	9.20	**<0.001**	2.65	0.461
Relationship	0.90	0.842	−9.10	0.100	−9.43	**0.004**	18.56	**<0.001**	−8.08	**0.039**
Chemotherapy										
No	Reference		Reference		Reference		Reference		Reference	
Yes	−7.51	**0.036**	8.18	0.070	5.20	0.090	−6.98	**0.009**	7.22	**0.034**
Hormonal therapy										
No	Reference		Reference		Reference		Reference		Reference	
Yes	−3.44	0.167	13.92	**<0.001**	4.39	**0.036**	−1.72	0.342	7.19	**0.002**
Radiotherapy										
No	Reference		Reference		Reference		Reference		Reference	
Yes	3.73	0.320	−5.66	0.228	−2.03	0.512	0.71	0.791	−1.08	0.754
Type of breast surgery										
BCS	Reference		Reference		Reference		Reference		Reference	
BCS + ALND	−0.31	0.933	−7.23	0.123	−0.26	0.932	2.65	0.320	−0.64	0.855
Mastectomy	8.05	**0.047**	−0.81	0.874	2.38	0.477	0.46	0.876	0.09	0.981
Mastectomy + ALND	3.18	0.343	−7.62	0.078	−4.04	0.149	2.36	0.328	−6.64	**0.035**
Body image	0.27	**<0.001**	−0.36	**<0.001**	−0.39	**<0.001**	0.23	**<0.001**	−0.47	**<0.001**
Maladaptive scale	−0.14	0.685	0.31	0.487	0.68	**0.018**	0.13	0.595	0.26	0.431
Adaptive scale	0.12	0.480	−0.03	0.894	0.08	0.585	0.09	0.477	−0.02	0.885
Observations	652		631		797		800		720	
R^2^	0.112		0.150		0.191		0.148		0.215	

Bold indicates *P* < 0.05.

ALND, axillary lymph node dissection; BCS, breast-conserving surgery; BMI, body mass index.

Having a relationship compared to being single, undergoing a mastectomy (with and without ALND) compared to BCS, and a higher body image score were positively associated with sexual QoL of AYA breast cancer survivors. Higher body image was found to have a positive association with all domains, as detailed in Table 3.

Being married compared to being single is both positively and negatively associated with sexual QoL. Married AYA breast cancer survivors reported significantly lower enjoyment of sexual activity compared to single AYA breast cancer survivors. However, they scored significantly higher on sexual functioning compared to single AYA breast cancer survivors (Table 3).

## Discussion

The results of this study showed that long-term sexual QoL is still a prominent issue among AYA breast cancer survivors up to 20 years after diagnosis particularly in terms of sexual symptoms and decreased sexual enjoyment. Sexual QoL was significantly worse in AYA breast cancer survivors compared to (female) AYA survivors of various other types of cancer. This is in alignment with findings from prior research on (older) cancer survivors and studies assessing QoL at a shorter time interval after diagnosis, which have demonstrated an increased risk of sexual dysfunction among breast and gynaecological cancer survivors.^19^^,^^24^^,^^49^ In the current study, sexual QoL of AYA breast cancer survivors was significantly worse compared to female AYA genitalia cancer survivors, corresponding with a recent systematic review.^19^ Additionally, there was no significant difference between sexual QoL of AYA breast cancer survivors and female AYA germ cell cancer survivors, aligning with a study demonstrating comparable QoL between breast and ovarian cancer survivors.^50^ The significant differences in sexual QoL between AYA breast cancer survivors and (female) AYA survivors of other cancer types might lay in the fact that AYA breast cancer survivors received more systemic therapy and radiotherapy compared to AYA survivors of other cancer types. Additionally, breast cancer survivors who have permanently become postmenopausal generally do not qualify for hormone replacement therapy after chemotherapy which contrasts with survivors of other cancers.^51^ This can all induce different sexual side-effects such as vaginal dryness, reduced libido, and change in body composition, which significantly impair sexual functioning and QoL.^52^, ^53^, ^54^, ^55^, ^56^, ^57^, ^58^ Furthermore, for many women, breasts are an important sexual feature as they represent femininity and sexuality.^59^ Therefore, breast cancer survivors experience more sexual QoL challenges than female survivors of other cancer types that do not involve the breasts.

In this study, age, time since diagnosis, relationship status, educational level, chemotherapy, hormonal therapy, type of surgery, body image, and maladaptive coping were all associated with sexual QoL of AYA breast cancer survivors. The identified factors such as chemotherapy, hormonal therapy, type of surgery, and body image correspond with findings from previous published literature that examined sexual health and functioning over shorter follow-up periods. More intense treatments, such as hormonal therapy and chemotherapy, are most frequently associated with sexual dysfunction.^24^^,^^33^^,^^38^^,^^39^ An older age and longer time since diagnosis were associated with poorer sexual QoL outcomes in the current AYA breast cancer cohort (18-39). This might be explained by the fact that AYA breast cancer survivors, who are older at the time of their diagnosis, might have more often a wish for children. Having a wish for children is associated with higher levels of reproductive concerns, which might influence overall sexual QoL.^38^^,^^60^ Another interesting and relatively new association found in this study is between maladaptive coping and sexual QoL of AYA breast cancer survivors. This aligns with a recent study focusing on an older breast cancer cohort.^61^

A somewhat unexpected finding from the current study is that undergoing a mastectomy compared to undergoing BCS is positively associated with sexual QoL, which contrasts with prior research.^33^^,^^62^^,^^63^ An explanation might be that almost all AYA breast cancer survivors who underwent a mastectomy also underwent a reconstruction, which is associated with improved sexual health.^64^ Another explanation might be the fact that patients undergoing a BCS almost always undergo additional radiotherapy to the breast, which can cause long-term side-effects such as pain and fibrosis of the breast and can negatively impact HRQoL, particularly among younger survivors.^57^^,^^65^, ^66^, ^67^ Additionally, women who have undergone BCS may tend to think more about cancer recurrence causing fear, which may influence their overall HRQoL and, subsequently, their sexual QoL.^68^

### Strengths and limitations

From previous literature, it is known that AYA cancer survivors experience ongoing problems with sexual functioning up to 2 years after diagnosis. However, there is limited knowledge regarding long-term sexual QoL.^37^^,^^69^ One of the major strengths of this study is the large sample size and the long-term sexual QoL data (5-20 years after initial diagnosis) that was available from the SURVAYA study.^2^ Another important strength of this study is the availability of various clinical and demographic variables from both the SURVAYA dataset and NCR. This enabled the examination of a wide range of possible associations.

One limitation of the current study is that no baseline measurement (before diagnosis and treatment) of sexual QoL was available; therefore, it was not possible to examine sexual QoL over time. In future research, it may be valuable to add a time component to the regression models as it is known that HRQoL changes over time.^70^ Furthermore, information on (types of) breast reconstruction from the NCR is available since 2011, and therefore it was not possible to include this in the regression models. As it is known that undergoing a breast reconstruction has an impact on QoL, further research including type of breast reconstruction is necessary.^63^^,^^71^ Another limitation of this study is that both physical and psychosocial comorbidities were not included in the analysis although they have a significant impact on (sexual) QoL.^44^^,^^72^^,^^73^ Unfortunately, data on cultural diversity were lacking, limiting the generalizability of the findings across different cultural contexts. Previous literature highlights the significant impact of cultural characteristics on sexuality and sexual function. Hence, it is essential to consider these aspects in future research.^74^, ^75^, ^76^ Moreover, it is important to note that only primary treatment details were available from the NCR. Therefore, it was not known exactly how hormonal treatment was defined (e.g. luteinizing hormone-releasing hormone (LH-RH) plus tamoxifen or an aromatase inhibitor) and for how long breast cancer patients were on this regimen, hypothesized to be 5 years or longer for most of the patients depending on the age at diagnosis (35 years; 10-year tamoxifen in combination with LH-RH was not unusual). Lastly, a significant limitation of this study is the absence of data on ovarian suppression and postmenopausal status, which are both critical factors affecting sexual QoL due to the estrogen deprivation.^77^ Although 50.7% of the AYA breast cancer survivors received endocrine therapy, details regarding their menstrual cycles or hormonal status at the time of evaluation were not available, unfortunately. For future research, collecting data on menstrual cycle is recommended to provide more comprehensive insights into sexual health outcomes of breast cancer survivors.

### Future perspective

Sexuality and intimacy are important aspects of AYA health care and sexual health should be discussed early in the treatment process.^35^^,^^78^ The results of this study demonstrated that AYA breast cancer survivors experience significantly more sexual QoL challenges compared to (female) AYA survivors of other cancer types. While discussing that sexual health is important for all AYA cancer survivors, those facing challenges related to sexuality, particularly breast cancer patients, might benefit from specialized interventions such as consultations with a sexologist. A recent literature review has highlighted the positive impact of psychological interventions on enhancing sexual health in women with breast cancer.^79^

Furthermore, this study identified different associations between patient and treatment characteristics and sexual QoL. Recognizing the different associations might enhance breast cancer health care as it can guide the development of already available targeted interventions such as coping mechanism interventions, body image workshops, psychological interventions, support regarding reproductive concerns, or relationship counselling. Additionally, the associations provide health care professionals with insights to better identify AYA breast cancer survivors at higher risk for sexual QoL challenges.

## Conclusion

AYA breast cancer survivors experience a significant decreased sexual QoL compared to (female) AYA survivors of other cancer types. Age, time since diagnosis, relationship status, educational level, chemotherapy, hormonal therapy, type of breast surgery, body image, and coping are all associated with sexual QoL of AYA breast cancer survivors. The results of this study will enhance targeted interventions to improve AYA breast cancer health care in the future.
