# Supplementary material for: Sexual quality of life of adolescents and young adult breast cancer survivors
Source: ESMO Open. 2024 Jan 27;9(2):102234. doi: 10.1016/j.esmoop.2024.102234 (PMC10937205; doi:10.1016/j.esmoop.2024.102234)
Supplement: Supplementary Appendix [file mmc1.docx]

**Appendix A**

Table S1. EORTC QLQ-SUV100 items

|  | **EORTC QLQ-SUV100 questions** |
| --- | --- |
| S102 | Has sexual activity been enjoyable for you? |
| S105 | Women only: Have you experienced a dry vagina during sexual activity? |
|  | *Sexual problems (symptom scale)* |
| S104 | Have you felt uncomfortable about the idea of being sexually intimate? |
| S107 | Have you avoided having sex? |
|  | *Sexual functioning (functional scale)* |
| S108 | Have you been interested in sex? |
| S109 | Have you been sexually active? |
|  | *Sexual problems when sexually active (symptom scale)* |
| S101 | Have you had problems being sexually intimate? |
| S103 | Have you had problems becoming sexually aroused? |
